# Successful echocardiography-guided medical management of severe early post-implant right ventricular failure in a patient with left ventricular assist device support: a case report

**DOI:** 10.1186/s13019-023-02368-1

**Published:** 2023-10-04

**Authors:** Shingo Kunioka, Osamu Seguchi, Tasuku Hada, Hiroki Mochizuki, Masaya Shimojima, Takuya Watanabe, Yasumasa Tsukamoto, Naoki Tadokoro, Satoshi Kainuma, Satsuki Fukushima, Tomoyuki Fujita, Hiroyuki Kamiya, Norihide Fukushima

**Affiliations:** 1 Department of Transplant Medicine, National Cerebral and Cardiovascular Center, Suita, Osaka Japan; 2 Department of Cardiac Surgery, National Cerebral and Cardiovascular Center, Suita, Osaka Japan; 3 Department of Cardiac Surgery, Asahikawa Medical University, Asahikawa, Hokkaido Japan; 4 Department of Nursing, Senri Kinran University, Suita, Osaka 565-0873 Japan

**Keywords:** Early post-implant right ventricular failure, Dilated cardiomyopathy, Left ventricular assisted device, Heart transplantation

## Abstract

**Background:**

Post-implant right heart failure (RHF) has been recognized as a crucial prognostic factor in patients receiving left ventricular assist devices (LVADs), and its management has long attracted attention from cardiologists and surgeons.

**Case presentation:**

This report described an 18-year-old female with acutely deteriorating heart failure due to dilated cardiomyopathy who underwent paracorporeal pulsatile-flow LVAD and developed early post-implant RHF. At postoperative day (POD) six, she was almost asymptomatic at rest on 2.5 mg/kg/min of dobutamine; however, the echocardiogram, performed as part of the daily postoperative care, revealed a severely enlarged right ventricle with a decompressed left ventricle, implying the development of post-implant RHF. Bolus infusion of saline and reduction of pump flow (6.0 L/min to 3.0 L/min) led to normalization of both ventricular shapes in 30 s, suggesting that RHF could be managed without surgical interventions. Milrinone was started on POD six, followed by sildenafil administration on POD seven. Fluid balance was strictly adjusted under the close observation of daily echocardiograms. Milrinone and dobutamine were discontinued on PODs 18 and 21, respectively. The patient was listed for a heart transplant on POD 40. Despite reduced right ventricular function (right ventricular stroke work index of 182.34 mmHg*ml/m^− 2^, body surface area 1.5 m^2^), she was successfully converted to implantable LVAD on POD 44 with no recurrence of post-implant RHF thereafter for four years.

**Conclusions:**

In post-implant RHF management, early detection, together with proper and prompt medical management, is crucial to avoiding any surgical intervention. Close observation of daily echocardiograms might be helpful in detecting subclinical RHF and is useful for post-implant medical management.

**Supplementary Information:**

The online version contains supplementary material available at 10.1186/s13019-023-02368-1.

## Background

Early post-implant right heart failure (RHF) is defined as the need for implantation of a temporary or durable right ventricular assist device (RVAD) and a left ventricular assist device (LVAD) for any duration, or failure to wean from inotropic or vasopressor support or inhaled nitric oxide within 14 days following LVAD implantation, or having to initiate this support within 30 days of implantation for a duration of at least 14 days [1]. Early post-implant RHF occurs in 20–50% of patients who receive LVAD implantation and is reported to increase postoperative morbidity and mortality [2–9]. Many studies have investigated the management of early post-implant RHF; however, none have provided a definitive management strategy according to the severity of this disease [2]. RVAD is one of the most definitive strategies for severe early post-implant RHF treatment; however, delayed introduction of RVAD might result in poor outcomes, and surgical interventions should be the last option [6, 10]. Therefore, both early detection of subclinically progressive RHF and accurate therapeutic decisions are crucial in caring for patients at early phase after LVAD implantation.

In our institution, a daily echocardiogram is routinely performed until two to three weeks post-operatively in all patients receiving LVAD implantation in order to detect any post-LVAD adverse events such as post-implant RHF, mediastinum hemorrhage, and aortic valvular insufficiency. An enlarged right ventricle, a collapsed left ventricle, and a distended inferior vena cava without any mediastinum hemorrhage are highly suspicious of RHF in LVAD patients, even if there are no clinical signs of RHF.

Here, we report a patient with early post-implant RHF after paracorporeal pulsatile LVAD implantation, identified as a subclinical adverse event by routine echocardiography prior to the development of clinical RHF and successfully managed by an adjustment of LVAD flow and appropriate medical management, without any invasive interventions.

## Case presentation

**Fig. 1:**
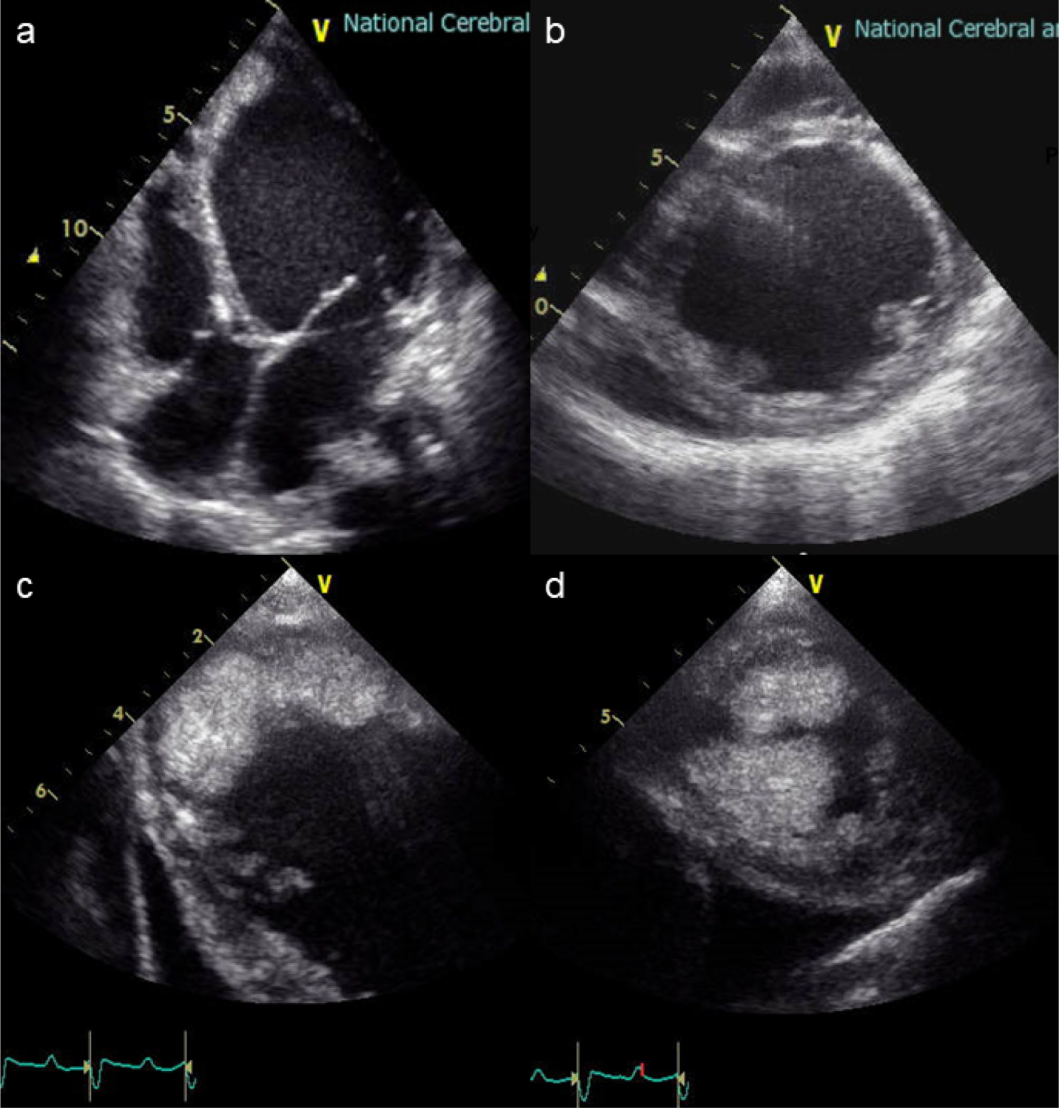
Transthoracic echocardiography findings before paracorporeal left ventricular assist device implantation. **(a,b)**: Echocardiography showed remarkable dilation of the left ventricle. The right ventricular volume was not excessive. **(c, d)**: Echocardiography showed a huge thrombus on the apical side of the left ventricle

An 18-year-old female with suspected end-stage dilated cardiomyopathy (DCM) was referred to our hospital for the presence of acutely deteriorating heart failure. Despite being on mechanical ventilation, inotrope infusion (dopamine 3.3 µg/kg/min, dobutamine 3.3 µg/kg/min), and intra-aortic balloon pump support, the patient developed severely decompensated heart failure complicated by multiple end-organ dysfunction [B-type natriuretic peptide, 2388.3 pg/ml; bilirubin, 2.1 mg/dL; aspartate aminotransferase (AST), 1090 U/L; blood urea nitrogen (BUN), 49 mg/dL; creatinine, 1.12 mg/dL]; therefore, implantation of paracorporeal ventricular assist device was considered for bridge to decision. Preoperative transthoracic echocardiography (TTE) revealed severely reduced left ventricular contraction with diffuse left ventricular wall thinning complicated by massive left ventricular thrombus (Fig. 1).

**Fig. 2:**
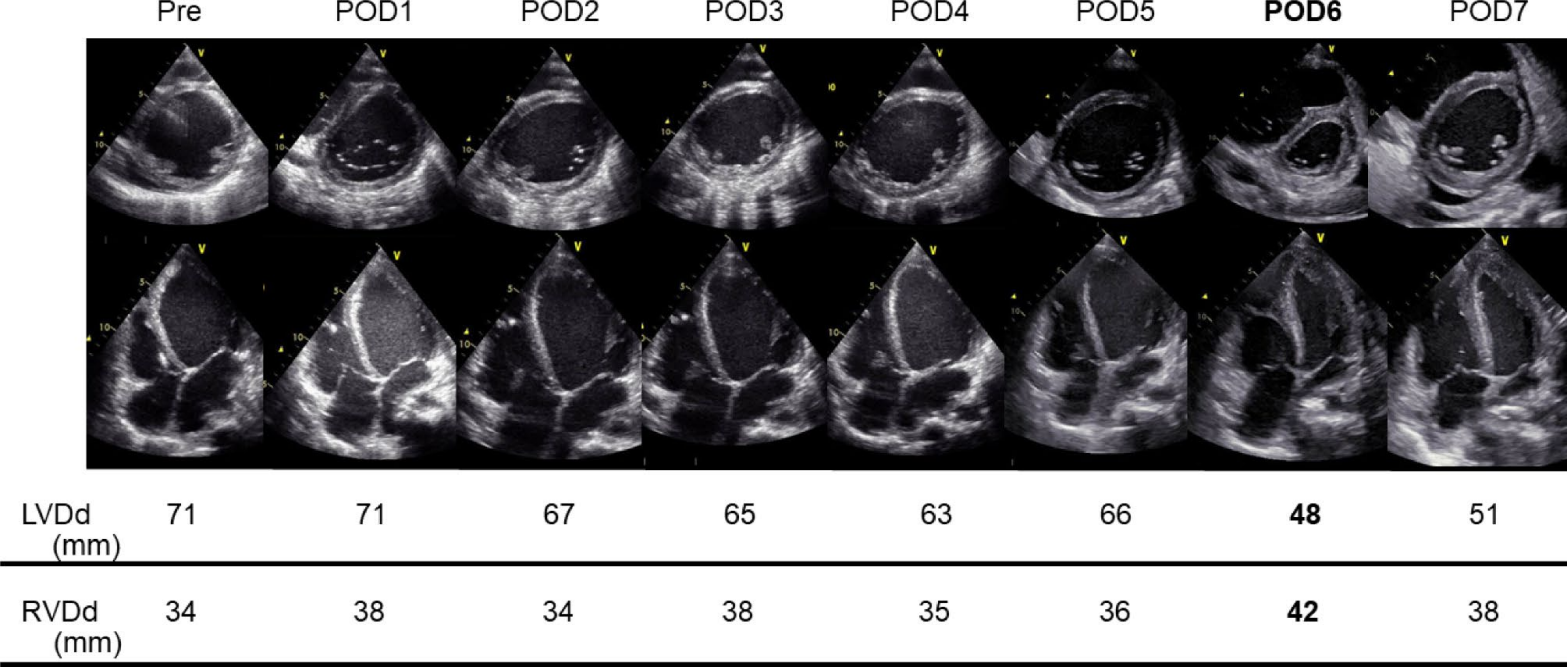
Daily echocardiographic findings during left ventricular assist device pump flow adjustment. These echocardiographic observations showed hemodynamic changes occurring at POD6, which revealed significant IVS shifting to the left ventricle. LVDd: left ventricular end-diastolic dimension, LVD: left ventricular diameter at end-systole, Pre: preoperative, POD: postoperative day

**Fig. 3:**
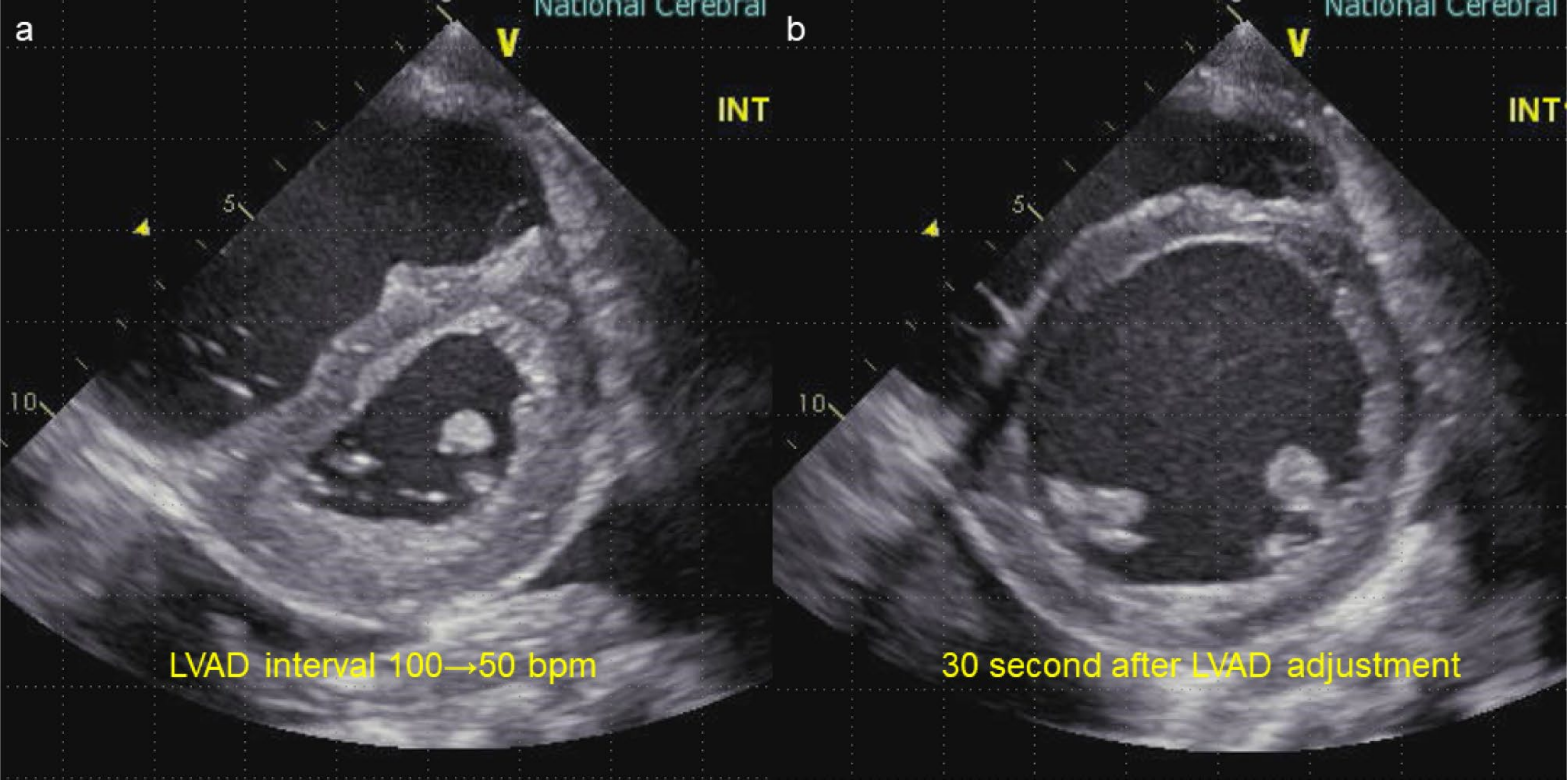
Echocardiographic findings during left ventricular assist device pump flow adjustment. **(a, b)**: This echocardiographic observation showed dramatic hemodynamic changes; the D-shape of the left ventricle recovered to an O-shape within 30 s

Right heart catheterization also showed severely elevated pulmonary capillary wedge pressure (PCWP, 30 mmHg), with a low right ventricular stroke work index (RVSWI, 298.13 mmHg*ml/m^− 2^), a low pulmonary arterial pulsatility index (PAPi, 0.87), and a low cardiac index (CI, 1.59 L/min/m2), which implied a potential risk for developing post-implant RHF (Table 1). Left ventricular thrombectomy and paracorporeal pulsatile-flow LVAD implant (Nipro-VAD®, Nipro Corporation, Japan) were successfully done, and the patient returned to the intensive care unit on 4.0 mg/kg/min of dobutamine and 15 ppm of inhaled nitric oxide. The early postoperative course was uneventful and the patient was extubated on postoperative day (POD) two. She was transferred to the advanced heart failure ward on POD four and daily echocardiogram revealed no signs of RHF while on 2.5 mg/kg/min of dobutamine infusion, and the mean arterial pressure was maintained approximately at 60–80 mmHg. On the morning of POD six, an echocardiogram revealed a severely enlarged right ventricle (RV) and decompressed LV combined with septal shift to the LV, suggesting the development of early post-implant RHF, with LVAD settings of 35% systole and a pump rate of 100 bpm (Fig. 2). Retrospectively, central venous pressure (CVP) was initially 15 to 20 mmHg on POD one, and it gradually decreased to below 10 mmHg on POD six. Therefore, early post-implant RHF due to insufficient preload to the RV was suspected, and we performed bolus saline infusion and adjusted the LVAD pump flow under the guidance of TTE findings. The Nipro LVAD pump flow could be changed by adjusting the percentage systole and interval rate; accordingly, the interval rate was adjusted from 100 to 50 bpm to reduce pump flow. Since one stroke volume of this device is approximately 50–70 ml, the predicted pump flow was estimated to have decreased from 6.0 L/min to 3.0 L/min if fully charged. In response to the reduced LVAD flow, both ventricular shapes were rapidly normalized in 30 s (Fig. 3, Supplementary video) based on TTE. We continued to administer 2.5 mg/kg/min of dobutamine and started 0.125 µg/kg/min of phosphodiesterase − 3 inhibitor (milrinone) and oral phosphodiesterase—5 inhibitor (sildenafil). Fluid balance was strictly adjusted under close observation of the daily echocardiogram, and milrinone and dobutamine were successfully discontinued on PODs 18 and 21, respectively, with no signs of post-implant RHF. The patient was listed as a candidate for a heart transplant on POD 40. Despite reduced RV function (RVSWI 182.34 mmHg*ml/m^− 2^), the patient was successfully converted to an implantable LVAD (HeartMate IITM, Abbott Laboratory, USA) on POD 44 without recurrence of RHF until she received a heart transplant four years after LVAD implantation (Table 2).

**Table 1:** Preoperative examination findings

Findings before LVAD implantation in patients with inotropic and IABP support	
Transthoracic cardiography	
EF (%)	12
LVDd/LVDs (mm)	72/67
IVSd/LVPWd (mm)	7/6
RVDd (mm)	26
IVC (mm)	15
AR/MR/TR/PR	Mild/trivial/mild/none
**Right heart catheterization**	
PCWP (mmHg)	30
mPAP (mmHg)	36
RAP (mmHg)	15
CI (L/min/m^2^)	1.59
PVR (WU)	2.51
PAPi	0.87
RAP/PCWP	0.5
RVSWI (mmHg*ml*m^2^)	298.13

AR: aortic regurgitation, CI: cardiac index, EF: ejection fraction, IABP: intra-aortic balloon pumping, IVC: inferior vena cava, IVSd: intraventricular septum diameter, LVAD: left ventricular assist device, LVDd: left ventricular end-diastolic dimension, LVDs: left ventricular diameter at end systolic, LVPWd: left ventricular posterior wall thickness, mPAP: mean pulmonary artery pressure, MR: mitral regurgitation, PAPi: pulmonary artery pressure index, PCWP: pulmonary capillary wedge pressure, PR: pulmonary regurgitation, PVR : pulmonary vascular resistance, RAP: right atrium pressure, RVDd: right ventricular end-diastolic diameter, RVSWI: right ventricular stroke work index, TR: tricuspid regurgitation

**Table 2:** Pre- and postoperative catheterization findings

	Pre-LVAD	POD 19 after paracorporeal LVAD implantation	HM II 1 M	HM II 1 Y	HM II 2Y	HM II 3Y
Number of rotations, rpm			8400	8600	8600	8600
PCWP, mmHg	30	5	2	2	2	4
mPAP, mmHg	36	14	11	7	8	8
RAP, mmHg	15	6	3	1	1	2
CI, L/min/m^2^	1.59	2.53	2.44	2.92	2.7	3.19
PVR WU	2.51	2.47	2.6	1.16	1.5	0.8
PAPi	0.87	1.83	3	11	14	6.5
RAP/PCWP	0.5	1.2	1.5	0.5	0.5	0.5
RVSWI, mmHg*ml/m^− 2^	298.13	182.34	256.84	253.91	266.2	262.19

CI: cardiac index, HMII: HeartMate II, LVAD: left ventricular assist device, mPAP: mean pulmonary artery pressure, PAPi: pulmonary artery pressure index, PCWP: pulmonary capillary wedge pressure, POD: postoperative day, PVR: pulmonary vascular resistance, RAP: right atrium pressure, RVSWI: right ventricular stroke work index

## Discussion and conclusions

Early post-implant RHF remains an important issue due to its association with high mortality and morbidity despite the presence of several studies investigating how to diagnose and manage it [2–9]. Given this background, the current case report highlights three practical suggestions regarding the diagnosis and management of early post-implant RHF in real-world LVAD therapy. First, routine daily echocardiogram regardless of clinical signs of hemodynamic compromise during early postoperative period is essential in detecting subclinical post-implant RHF as early as possible. Second, during the early postoperative period, appropriate pump setting followed by proper medical managements are crucial, as adjustment of pump setting directly affects ventricular geometry on a second-by-second basis. Third, development of RHF is multifactorial and RV function fluctuates depending on preload, afterload, RV contractile function and other factors [11, 12]. Therefore, it is still difficult to accurately predict the development of post-implant RHF based on preoperative clinical parameters; however, it is still possible to prevent post-implant RHF regardless of lower baseline RV function.

Echocardiography is one of the practical modalities for assessing hemodynamic conditions and LVAD-related adverse events after LVAD implantation. Despite difficulties in obtaining clear images in early phase after surgery, daily echocardiogram is routinely examined until two to three weeks after LVAD implantation regardless of clinical symptoms. During the echocardiography examination, inferior vena cava, dimensions of both LV and RV, tricuspid valve, mitral valve, and aortic valve are observed, if possible. Furthermore, the position of inflow cannula and velocity time integral of RV outflow are also examined to evaluate how LVAD works, if available. Pleural effusion of both lungs, and pericardial effusion are also investigated to detect any complications that would need surgical interventions. IVS shift is an effective parameter to detect early post-implant RHF as it can be obtained even in the early phase of the postoperative period and used to evaluate the balance of both ventricles, reflecting RV function without requiring detailed measurements [13–15]. In the current case, echocardiography was well visualized from POD one, and there was no sign of RHF until POD five. However, on POD six, both LV and RV morphologies completely changed to an enlarged RV combined with decompression of the LV, suggesting the development of post-implant RHF. At that time, the patient’s vital signs were all stable with no signs of hemodynamic compromise; therefore, our routine echocardiogram detected subclinical RHF before the development of symptoms of RHF such as renal or liver dysfunctions. Since appropriate RV geometry is essential to maximize RV function [16], our routine echocardiogram contributes to the early detection and resolution of subclinical RHF, which may be a key in preventing the development of subclinical, and symptomatic post-implant RHF.

Regarding pump setting in the early postoperative period, it should be timely changed depending on patients’ systemic conditions. During the very early postoperative period, such as PODs one to three; pump setting should be maximized to maintain enough blood supply to peripheral organs. Afterwards, pump setting generally decrease along with the decrease in fluid volume. When we reviewed the patient’s early postoperative course, CVP, which was initially 15 to 20 mmHg on POD one, gradually decreased to below 10 mmHg on POD six. In the setting of baseline vulnerable RV function in this patient, adequate preloading is important for treating subclinical RHF on POD six. Since dynamic fluid volume changes are often seen in patients receiving LVAD implantation, a closer follow-up using multiple modalities, including echocardiography and adjustment of pump setting combined with timely medical interventions is essential. Drastic changes of both RV and LV geometry in 30 s is a highly comprehensive presentation showcasing the deep interdependence between pump setting and post-implant RHF development.

To date, although various risk factors and risk-prediction models for post-implant RHF have been reported, no universal predictors for post-implant RHF have been established. Since the definition of post-implant RHF varied according to the studies and the development of post-implant RHF is multifactorial, including not only patient-related factors but also device-related factors, careful selection of the parameters is warranted in real-world clinical practice. We applied “the right ventricular risk score (RFRS)” reported by Matthews et al. to this case since the definition of post-implant RHF in that study included intravenous inotrope support for > 14 days, and the devices used in that study were mainly pulsatile pumps, both of which are similar to those used in the current case [4]. Furthermore, hemodynamic parameters were examined to estimate the native right ventricular function; right atrial pressure (RAP)/PCWP ≥ 0.63 and the need for preoperative ventilator support were independent predictors of post-implant RHF [5]. PAPi < 1.85 was also strongly associated with post-implant RHF [8], as were CI ≤ 2.2 L/min*m2, RVSWI ≤ 250 mmHg*ml*m-2, and severe preoperative RV dysfunction. The RFRS of this patient was 4.5, and RAP/PCWP, PAPi, CI, and RVSWI were 0.5, 0.87, 1.59 L/min/m2 and 298 mmHg*ml*m-2, respectively, all of these suggest mild to moderate risk for post-implant RHF. In such patients, it might be important to detect hemodynamic changes and take prompt action before early post-implant RHF becomes symptomatic. However, after conversion to axial-flow LVAD, despite the low RVSWI and RAP/PCWP ratio, the patient had been uneventfully supported for almost four years and successfully switched to transplantation after 1498 days of support with appropriate postoperative RHF management.

Recently, Impella RP (Abiomed, Danvers, Mass., USA) was reported to support the RV in patients with post-implant LVAD [17]. It might be a useful tool for patients who have subclinical or mild RVF and in whom RVAD might be excessive treatment.

In conclusion, early detection, together with both proper and prompt medical management, is crucial to avoiding surgical intervention in the management of post-implant RHF. Further, close observation of the echocardiogram might be helpful in detecting subclinical early post-implant RHF and for daily medication assessment.

### Electronic supplementary material

Below is the link to the electronic supplementary material.

Supplementary Material 1

## Data Availability

Raw data of the participant are not publicly available to preserve the individual’s privacy under the General Data Protection Regulation.

## List of abbreviations

AST: aspartate aminotransferase
CI: cardiac index
CVP: central venous pressure
LVAD: left ventricular assist device
POD: postoperative day
PCWP: pulmonary capillary wedge pressure
RAP: right atrial pressure
RHF: right heart failure
RV: right ventricle
RVAD: right ventricular assist device
RFRS: right ventricular risk score
RVSWI: right ventricular stroke work index
TTE: transthoracic echocardiography
